# SARS-CoV-2 breakthrough infections during the second wave of COVID-19 at Pune, India

**DOI:** 10.3389/fpubh.2022.1040012

**Published:** 2023-01-12

**Authors:** Prakash P. Doke, Suhas T. Mhaske, Gauri Oka, Ruta Kulkarni, Vrishali Muley, Akhilesh Chandra Mishra, Vidya A. Arankalle

**Affiliations:** ^1^Department of Community Medicine, Bharati Vidyapeeth (Deemed to be University) Medical College, Pune, India; ^2^Department of Communicable Diseases, Interactive Research School for Health Affairs (IRSHA), Bharati Vidyapeeth (Deemed to be University), Pune, India; ^3^Department of Microbiology, Smt. Kashibai Navale Medical College, Pune, India

**Keywords:** SARS-CoV-2, COVID-19, vaccination, breakthrough infection, variants, neutralizing antibodies

## Abstract

Breakthrough infections following SARS-CoV-2 vaccination remain the global concern. The current study was conducted during the second wave of COVID-19 (1st March−7th July 2021) in Pune, India, at two tertiary care hospitals. Of the 6,159 patients diagnosed as COVID-19, 372/2,210 (16.8%) were breakthrough infections. Of these, 81.1 and 18.8% received one or two doses of Covishield or Covaxin, respectively. Of note, 30.7% patients were with comorbidities, hypertension being the commonest (12.44%). The majority of infections were mild (81.2%). Forty-three patients with breakthrough infections were hospitalized with severe (*n* = 27, 62.8%) or moderate (*n* = 16, 37.2%) disease. The receptor binding domain (RBD) sequences from vaccinated (*n* = 126) and non-vaccinated (*n* = 168) samples were used for variant analysis. The delta variant was predominant followed by kappa in both vaccinated and non-vaccinated groups. Viral load (qRT-PCR) was not different among these categories. Full-genome comparisons of sequences in relation to vaccination status did not identify any mutation characteristic of the vaccinated group. Irrespective of the number of doses, neutralizing antibody titers (PRNT50) during the first week of clinical disease were higher in the vaccinated patients than the unvaccinated category. In conclusion, though not completely, SARS-CoV-2 vaccines used for country-wide immunization did reduce disease severity among the individuals without any comorbidity by inducing rapid immune response against distinctly different delta and kappa variants. The utility against emerging variants with further mutations need to be carefully examined.

## Introduction

COVID-19 pandemic initiated in 2019 continues to affect global population. The pandemic is characterized by rapid development of vaccines employing conventional and novel platforms and immediate use for immunization of global population. Development of herd immunity was anticipated to control the spread of the virus. In reality, the virus underwent specific mutations in different countries leading to emergence of variants with variable transmission potential. Observed reinfections and breakthrough infections among vaccinated individuals raised questions about the efficacy of different vaccines. Soon, efficacy was measured by reduced hospitalizations and mortality. So far, breakthrough infections have been reported with all the vaccines used viz Germany (Pfizer vaccine) (1), UK (Moderna and Pfizer vaccines) (2), USA (Moderna and Pfizer vaccines) (3–8), Israel (Pfizer vaccine) (9), Qatar (Moderna and Pfizer vaccines) (10, 11) and India (Covaxin and Covishield) (12–17).

As of 31^st^ August 2022 more than 0.5 billion infected and more than 6 million death cases have been reported worldwide (18). In India, more than 44 million infected cases and 0.5 million deaths have been reported (19). A national COVID-19 vaccination program was initiated on 16^th^ January 2021 for health care workers and subsequently extended to other populations as per priority. COVISHIELD, the chimpanzee adenoviral vectored vaccine with full length SARS-CoV-2 spike insert manufactured by Serum Institute of India Pvt Ltd (SIIPL) and COVAXIN, an indigenous, whole virus-inactivated vaccine manufactured at Bharat Biotech International Ltd (BBIL) were used, COVISHIELD contributing to ~80% immunizations (20). COVISHIELD is the Indian version of AZD1222 manufactured by AstraZeneca (Vaxzevria). The present comprehensive study attempting to assess early breakthrough infections was initiated 4.25 months after the first use of vaccine administered as two doses, 4 weeks apart, in the Indian population.

## Methods

### Ethical considerations

This study was approved by the institutional ethics committee (DCGI Reg. No. ECR 518/Inst/MH/2014/RR-17) vide REF: BVDUMC/IEC/03, Date- 30/03/2021. The research coordinators obtained telephonic verbal consent for getting history and then for collection of blood from all the participants.

### Clinical specimens

During 1^st^ March to 7^th^ July 2021, SARS-CoV-2 RNA positive NPS samples were collected from two tertiary care hospitals in Pune, India, namely Bharati Hospital (Total 2,454, 188 vaccinated, 2,266 non-vaccinated) and Smt. Kashibai Navale Hospital (Total 83, 74 vaccinated, 9 non-vaccinated).

### RNA extraction, PCR and sequencing

Viral RNA was extracted from 566 NPS (198 vaccinated, 368 non-vaccinated) samples using QIAamp viral RNA mini kit (Qiagen CA, USA) and cDNA was synthesized using high-capacity reverse transcription kit (Thermofisher Scientific, MA, USA) using reverse primer (5′ GTCTGAGTCTGATAACTAGCGC 3′) of spike gene region (23,595–23,574). The Nested PCR approach was used for the amplification of the RBD region of SARS-CoV-2. The 1^st^ PCR was set using Forward primer (5′ GTAATGATCCATTTTTGGGTG 3′) of the spike region (21,969–21,989) and Reverse primer (5′ GTCTGAGTCTGATAACTAGCGC 3′) of the spike region (23,595–23,574) whereas nested PCR was set using forward RBD-F (5′ AATATTACAAACTTGTGCCCT 3′) and reverse RBD-R (5′ AACAGTTGCTGGTGCATGTA 3′) primers. PCR products of Nested PCR positive samples (~600 bp) were gel purified and sequenced from both the ends using Big Dye terminator cycle sequencing kit (Thermofisher Scientific, MA, USA). RBD sequences were confirmed by BLAST (www.ncbi.nlm.nih.gov/BLAST). The forward and reverse sequences were aligned using MEGA 10 software to obtain the consensus sequence. Partial RBD sequences were submitted to GenBank database at www.ncbi.nlm.nih.gov (Supplementary Table 1).

### Whole genome sequencing and analysis

The full genomes of SARS-CoV-2 from vaccinated (*n* = 2, GenBank Acc No-MZ574051 and MZ574056) and non-vaccinated (*n* = 4, MZ574052, MZ574053, MZ574054 and MZ574055) patients were obtained as described earlier (21). In addition, full genome sequences deposited in GISAID database (https://gisaid.org/) were also used. These included 12 sequences from the vaccinated (GISAID ID: EPI_ISL_2017767, EPI_ISL_2405140, EPI_ISL_2405143, EPI_ISL_4988039, EPI_ISL_6930117, EPI_ISL_6930069, EPI_ISL_4955680, EPI_ISL_4955729, EPI_ISL_4955689, EPI_ISL_4955720, EPI_ISL_4955719, and EPI_ISL_4955705) and 9 sequences from the non-vaccinated (EPI_ISL _4988615, EPI_ISL _4988592, EPI_ISL _4988699, EPI_ISL _4955733, EPI_ISL _4988698, EPI_ISL _4988822, EPI_ISL _4988741, EPI_ISL _4988717, and EPI_ISL _4988828) patients diagnosed during the same time in different regions of India.

### SARS-CoV-2 IgG ELISA

Fifty two serum samples collected from patients with breakthrough infections were tested for the presence of anti-SARS-CoV-2 IgG antibodies using SCoV-2 Detect™ IgG ELISA (InBios International, Inc., USA) as per the manufacturer's protocol.

### SARS-CoV-2 PRNT

SARS-CoV-2 PRNT was performed for the same 52 serum samples in BSL3 laboratory using Vero CCL81 cells procured from ATCC and maintained in minimum essential medium (MEM; Gibco, Waltham, MA, USA) with 10% fetal bovine serum (FBS; Gibco, Waltham, MA, USA) and antibiotics, including penicillin–streptomycin (100 μg/ml; Thermo Fisher Scientific, Waltham, MA, USA) at 37°C in a humidified incubator with 5% carbon dioxide (CO2). For the assay, cells were seeded at a density of 1 x 10^5^ cells/well in a 24-well plate, 1 day prior to infection. Serum samples diluted 1:5 in MEM containing 2% FBS and antibiotics, were subjected to heat inactivation followed by 4-fold serial dilutions. Each dilution was mixed with equal volume of 20–40 PFU of SARS-CoV-2, followed by incubation at 37°C. After 1 h, each virus-serum mixture was added in duplicate wells of the seeded 24-well plate, and incubated for 1 h, followed by addition of overlay medium containing MEM, 1% carboxymethyl cellulose (Aquacide-II, Merck, Calbiochem-Merck, San Diego, CA, USA), 2% FBS and antibiotics. At 5 days post infection, cells were fixed using 3.7% formaldehyde and stained using 1% crystal violet (Sigma-Aldrich., Inc, Saint Louis, MO, USA). Plaques were counted and PRNT_50_ titer was determined using the standard logistic regression method. Samples with PRNT_50_ titer >10 were considered positive for neutralizing antibodies.

### Statistical analysis

For the comparisons of proportions, Chi Square test was performed. For other analyses, *T* test (non-parametric, Mann-Whitney) was used, *p* < 0.05 was considered as statistically significant.

## Results

### Patients, clinical samples, viral RBD RNA positivity and viral load

During the study period, naso-pharyngeal-swabs (NPS) from 16,646 suspected COVID-19 patients were tested for SARS-CoV-2 RNA and 6,159 were found to be positive. Among the positives, information about vaccination was obtained from 2,210 and 372 reported history of (H/O) vaccination. Of these, 184 vaccinated patients consenting for providing blood sample within 1 week were included in the study. Table 1 describes patient characteristics. There was no difference when data sets for 372 and 184 patients were compared (*p* > 0.05 for all). Among vaccinated persons, hypertension was the commonest comorbidity (12.44%), followed by duo of hypertension plus diabetes (9.33%) and diabetes (7.51%). Twenty-eight (7.2%) patients developed severe disease, 18 (4.7%) had a moderate infection while mild infection was recorded in 321 (81.2%). Such information was not collected from the non-vaccinated patients.

**Table 1:** Characteristics of the patients providing H/O vaccination and the subset from whom nasopharyngeal swabs were obtained.

**Parameter**	**Patients with H/O vaccination**	**No of patients included in the study** **(NPS screened for viral RNA)**	***p*-Value**
Total no of patients	372	184	
Males: Females	215:157	104:80	0.77
Age <50:>50 years	210:162	106:78	0.79
Comorbidities^*^	113/367 (30.7%)	63/177 (35.5 %)	0.26
Severe disease	28/367 (7.6 %)	27/177(15.2 %)	0.009
Moderate disease	18/367 (4.9 %)	16/177 (9 %)	0.093
Mild disease	321/367 (87.4%)	152/177 (85.8 %)	0.61
No of recipients (1 dose)	200^**^	90^**^	0.96
Covishield	156 (78%)	70 (77.7 %)	
Covaxin	44 (22%)	20 (22.2 %)	
No of recipients (2doses)	103^**^	52^**^	0.43
Covishield	90 (87.3 %)	43 (82.6 %)	
Covaxin	13 (12.6 %)	9 (17.3 %)	

* Diabetes and hypertension either singly or in combination.

** Information about the type of vaccine was available for 303.

**Figure 1:**
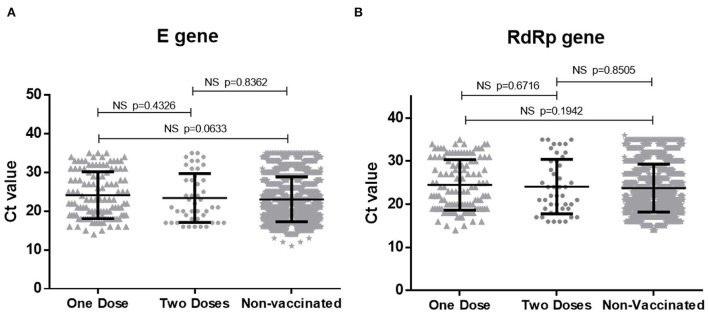
Comparison of Ct values for **(A)** E gene and **(B)** RdRp gene of SARS-CoV-2 in vaccinated and non-vaccinated COVID-19 patients. NS, non significant.

NPS specimens collected from 184 vaccinated patients at the time of diagnosis were subjected to PCR for RBD gene and 126 (68.4%) scored positive. Of the positives, 84 (66.6%) and 42 (33.3%), respectively received one and two vaccine doses, respectively (*p* < 0.001). During the same period, 168 RBD-PCR positive NPS specimens from non-vaccinated COVID-19 patients were sequenced for variant analysis. PCR positivity at the time of first diagnosis was higher in unvaccinated than the vaccinated patients (*p* < 0.01). Comparison of Ct values as a measure of viral load documented no difference among vaccinated and non-vaccinated patients (*p* > 0.05; Figure 1).

### SARS-CoV-2 variants among vaccinated and non-vaccinated COVID-19 patients

As evident from Table 2, infection with the wild (Wuhan) virus was low in both patient categories while delta variant was predominant followed by the kappa variant. The UK variant (alpha) was detected in one patient from non-vaccinated category. Additional infrequent mutations included V382L and N440K.

**Table 2:** Variant analysis^*^ in COVID-19 patients with or without H/O vaccination.

**No of vaccine doses (No of recipients)**	**Wild (Wuhan)**	**kappa**	**Delta**	**Alpha**
One dose (84)	2 (2.3%)	26 (30.9%)	56 (66.6%)	0
2 doses (42)	0	13 (30.95%)	29 (69.05%)	0
None (168)	4 (2.38%)	41 (24.4%)	117 (69.65%)	1(0.59%)

* Six (3 each in 1 and 2 doses recipients) sequences exhibited V382L mutation while 3 among the non-vaccinated group exhibited N440K mutation.

### Variability in the detection frequency of variants over time during the study period

Previously, we showed the emergence of kappa variant during the earlier phase of the second wave followed by co-circulation with delta variant and subsequent complete replacement by the delta variant (21), Therefore, we compared detection rates of the variants in vaccinated and non-vaccinated patients infected during different months (Table 3). Except for the kappa variant, the presence of other variants was similar during the study months. In April, kappa variant was more frequent in vaccinated patients than the non-vaccinated category (*p* < 0.01). In May, delta variant replaced the kappa variant in the non-vaccinated group while 15.8% vaccinated patients were infected by this variant.

**Table 3:** Month-wise detection of SARS-CoV-2 variants in vaccinated and non-vaccinated patients.

**Month of NPS**	**Partially/Fully vaccinated (*n* = 126)**			**Non-vaccinated (*n* = 168)**				
**collection**	**No of variants identified/No tested**			**No of variants identified/No tested**				
	**Kappa**	**Delta**	**No mutation**	**Alpha**	**Kappa**	**Delta**	**No mutation**	**N440 K**
Mar-21	10/13 (76.9%)	1/13 (7.6%)	2/13 (15.3%)	0/33	27/33 (81.8%)	1/33 (3%)	3/33 (9%)	2/33 (6%)
Apr-21	19/37 (51.3%)	18/37 (48.6%)	0/37	1/54 (1.8%)	15/54 (27.3%)	36/54 (67.9%)	1/54 (1.8%)	1/54 (1.8%)
May-21	10/63 (15.8%)	53/63 (84.1%)	0/63	0/74	0/74	74/74 (100%)	0/74	0/74
Jun-21	0/9	9/9 (100%)	0/9	0/7	0/7	7/7 (100%)	0/7	0/7
Jul-21	0/3	3/3 (100%)	0/3	0	0	0	0	0

### Whole SARS-CoV-2 genome sequences derived from vaccinated and non-vaccinated COVID-19 patients

For sequence analysis, 6 full genomes of delta variant sequenced by us were used (21). Of these, two represented vaccinated and 4 were non-vaccinated patients. In addition, sequences of the viruses from patients from different parts of India with (*n* = 12) or without (*n* = 9) vaccination and collected during the same period were included for the analysis. The SARS-CoV-2 whole genome sequence of Wuhan origin was used as reference sequence (GenBank accession number, NC_045512). We did not identify a single amino acid mutation related to vaccination.

### Antibody response during breakthrough infections

**Figure 2:**
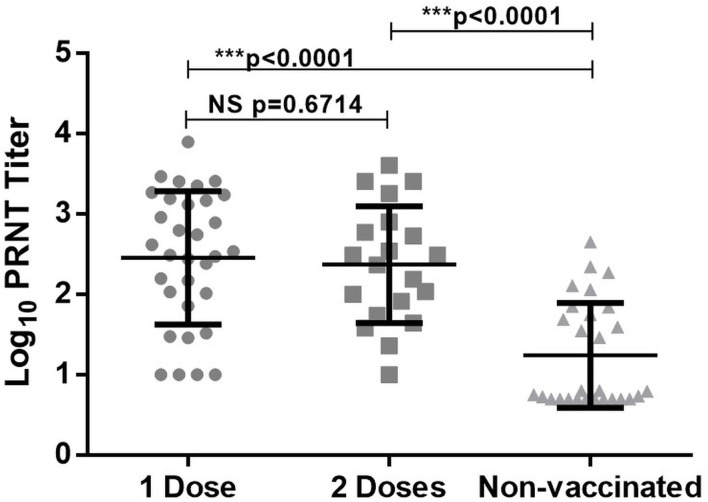
PRNT_50_ titer in breakthrough infection (1 or 2 doses of vaccine) and non-vaccinated individual. ***Denotes high statistical significance with *P* value < 0.0001, NS denotes statistically non significant.

Previously we had observed low/no antibodies in COVID-19 patients tested during the first week post-onset of clinical symptoms (22, 23). To assess if infection among vaccinated individuals leads to early antibody response, serum samples from 52 patients were screened within 5 days of the onset of clinical symptoms. Of the 32 patients receiving one dose of the vaccine, 29 (90.6%) and 28 (87.5%) were reactive in ELISA and PRNT respectively. Among fully vaccinated patients, ELISA positivity was 100% (20/20) and one patient lacked neutralizing antibodies (19/20, 95%). As compared to the non-vaccinated patients (55 ± 96), neutralizing antibody titres in vaccinated patients with 1 dose (988 ± 1,538, *p* < 0.0001) or 2 doses (734 ± 1,112, *p* < 0.0001) of the vaccines were significantly higher (Figure 2). The titers were independent of the number of doses received (*p* = 0.67).

### Severity among vaccinated individuals

During the study period, 43 breakthrough infections requiring hospitalization were identified. These included 27 patients with severe disease (62.8%). Of note, there was no mortality. In accordance with predominant use of COVISHIELD, 37 were administered Covishield (86.04%) while only six received COVAXIN (13.95%, age 53–76 years, all males) while. Variant identification was done for 8 patients; 7 were infected with delta variant and 1 with the kappa variant. With the small number with Covaxin, no difference was observed when both the vaccines were compared (Table 4).

**Table 4:** Breakthrough infections requiring hospitalization and vaccine type.

**Parameter**	**Vaccine type**		**Total**
	**COVAXIN**	**COVISHIELD**	
No of breakthrough infections	6	37	43
Median Age (range), years	53–76	43–72	43–76
Males: females	6:0	27:10	33:10
No receiving 1 dose	4 (66.7%)	34 (91.9%)	38 (88.4%)
No receiving 2 doses	2 (33.3%)	3 (8.1%)	5 (11.6%)
Comorbidities	5 (83.3%)	24 (64.9%)	29 (67.4%)
No with severe disease	5 (83.3%)	17 (45.9%)	27 (62.8%)
No with moderate disease	1 (16.7%)	15 (40.5%)	16 (37.2%)
Variant type/ No sequenced	Delta (*n* = 3)/3	4 Delta/5 1 Kappa/5	7 Delta, 1 Kappa

## Discussion

We provide detailed analysis of 184 breakthrough infections among Covishield and Covaxin recipients diagnosed at two tertiary care hospitals from Pune, India. In view of the initiation of vaccination on 16^th^ January 2021 and the study period of 4.25 months starting from March 2021, it is clear that the vaccine efficacy was naturally tested against the highly transmissible delta variant with global spread and kappa variant with restricted transmission (24). History of vaccination by 372/2210 COVID-19 patients diagnosed during this period estimated that 16.8% patients represented breakthrough infections. A retrospective test-negative case-control study (25) involving 28,342 vaccinees from 24 Indian cities identified 5.07% breakthrough infections, 80 (0.28%) requiring hospital admission and 3 (0.01%) needing intensive care units. The proportion of breakthrough infections for different vaccines varied from 5.07 to 19% during the delta (12–14, 25) and 71–83% during the Omicron wave (26–28). We did not study breakthrough infections during the third wave.

As we could study 184/372 (49.4%) patients providing H/O vaccination, patient characteristics were compared among these patient groups. Except for disease severity, all the other parameters were similar (*p* = 0.26–0.96). As almost all the hospitalized patients with moderate / severe disease were captured in the study, the proportion included in the study was higher. Currently, lack of hospitalization and death are considered as the pointers for successful COVID-19 vaccinations. In our cohort, no death was recorded and all the patients with severe disease had at least one comorbidity. Clearly, the vaccine did help in reducing disease severity among individuals without comorbidities. Higher proportion of COVISHIELD recipients developing breakthrough infection reflects ~80% use of this vaccine in India and not connected to the vaccine type. Both the vaccines employing either the whole virus or spike protein contributed to breakthrough infections. Similar to other reports, significant breakthrough infections were noted even after receiving 2 doses (29) emphasizing need for a third dose or an early booster. Based on our study it may be surmised that the population without any comorbidity may benefit from the current vaccines. A larger sample size will be required to identify further subgroups with or without benefits.

As we were monitoring variants during the pandemic in the same setting (21), we could compare infecting variants during the second in relation to vaccination status (Table 2). The variant of concerns (VOCs) circulating in Brazil and South Africa were not detected with only 1 non-vaccinated patient infected with the UK strain. With the limited samples analyzed, mutations under immune pressure, including K417N signifying Delta plus variant were not detected. In fact, we did not detect any amino acid mutation that could be attributed to the use of vaccine. Thus, breakthrough infections were mainly attributed to kappa variant earlier and predominated by delta variant later. Whether complete replacement of kappa with the delta variant during the second wave reflected higher transmissibility of the delta variant remains unanswered. Importantly, Kappa and Delta variants infected both vaccinated and non-vaccinated individuals to similar extent questioning protection offered by the vaccines against emerging variants. Here, an important observation by Mlcochova et al. is noteworthy. In lung Calu-3 cells bearing endogenous receptors, replication of the Delta variant was significantly higher than the Kappa variant as evidenced by qRT-PCR and infectivity assays. Replication of both these variants was higher than the Wuhan virus with D614G mutation (30). Explosive increase in the number of fatalities during 21^st^ June to 14^th^ August 2021 in Iran correlated with the emergence of Delta plus variant (31). The continued dominance of the delta variant in India and replacement with Delta plus in Iran may represent differential evolution of the virus in different populations with varied immune responses. Taken together, currently available vaccines are not likely to protect against emerging variants with mutations that modify immune-dominant epitopes on the spike protein.

Our study was limited to the second wave. However, the third wave caused by the Omicron variant and the subvariants detected first in Africa needs to be specifically mentioned. Omicron variants, especially BA2 with three unusual biologic properties of high transmissibility, weaker virulence and evasion from the neutralizing antibodies generated by available vaccines as well as natural infection with the earlier variants have led to significant rise in the number of cases globally (32). Due to mild disease and recovery, diagnosis was not done in the majority of the cases in India and hence it is difficult to truly assess the number of infections by Omicron variant. Approximately 4 months after the start of the Omicron wave, emergence of BA4/5 variants causing the fifth wave was identified (33). We did record BA1, BA2 initially and BA4/5 later during the third wave at Pune, India (our unpublished observations).

The observed high rate of breakthrough infections with different variants could be due to the choice of mutation prone spike antigen derived from the Wuhan strain as a vaccine candidate. The introduction of critical mutations, altering conformation of immunodominant epitopes in the emerging variants reduces efficacy of the currently used vaccines. Notably, certain mutations in the spike protein facilitate rapid entry and replication of the virus (30, 31). For the protection of global population in general and those with comorbidities in particular, additional doses/early boosters and improved vaccines with broader neutralization potential are indeed necessary.

Another possible benefit of vaccination would be lower replication of the virus due to rapid and early rise in immune response during breakthrough infections. This is likely to result in reduced replication of the virus leading to lower transmission. In contrast to the reported lower viral load in breakthrough infections among mRNA vaccine recipients (34), we did not observe any difference in patients receiving 1 or 2 doses of the vaccines. Thus, vaccination may not have helped in lower viral transmission by vaccinated patients during the second wave of the disease. A recent study documented that genomic viral loads were similar irrespective of vaccination (35). However, infectious virus could be detected for a longer time among non- or partially vaccinated patients than in the fully vaccinated individuals. In the light of viral load estimations by q-RT-PCR by most studies, this is indeed a significant observation suggesting positive role of vaccination in lowering further transmission.

Quantitation of neutralizing antibodies by a live virus neutralization test has led to significant information. During the first week of clinical disease, neutralizing titers were significantly higher among the vaccinated patients, irrespective of the number of doses (Figure 2). It is satisfying to note that memory immune cells generated by the vaccines based on Wuhan strain were able to rapidly boost neutralizing antibody titers after encountering a different variant. It may be assumed that vaccination will be able to induce rapid immune response against emerging variants, unless major epitopes are modified.

Though our study involves samples collected only from two tertiary hospitals, it does represent the situation in the Pune city. The current study demonstrated the variants of SARS-CoV-2 leading to breakthrough infection in the second wave of COVID-19 in Pune and underline the importance of surveillance to identify the circulating variants and breakthrough infection assisting to monitor the surge of vaccine escape variants of concern.

In conclusion, our study during the second wave shows that though not against infection, immunization with COVID-19 vaccines in India did reduce severity and associated hospitalizations among individuals without comorbidities. Proportion of infecting variants was similar irrespective of vaccination status. Patients with breakthrough infections developed earlier and higher neutralizing antibodies probably helping in reduced severity. For protection against newer variants, improved vaccines need to be developed that produce cross-variant humoral and cellular immunity.

## Data availability statement

The datasets presented in this study can be found in online repositories. The names of the repository/repositories and accession number(s) can be found in the article/Supplementary material.

## Ethics statement

The studies involving human participants were reviewed and approved by Bharati Vidyapeeth (Deemed to be University) Medical College. Written informed consent was not provided because the research coordinators obtained telephonic verbal consent for getting history and then for collection of blood from all the participants.

## Author contributions

PD and VA: concept, study design, and supervision. SM and RK: performance of experiments, data analysis, and interpretation. GO and VM: recruitment of patients and clinical management. SM, PD, and VA: writing of the manuscript. AM: review of the results and the manuscript. All authors read and approved the final manuscript.
