# Supplementary material for: SARS-CoV-2 breakthrough infections during the second wave of COVID-19 at Pune, India
Source: Front Public Health. 2023 Jan 12;10:1040012. doi: 10.3389/fpubh.2022.1040012 (PMC9877521; doi:10.3389/fpubh.2022.1040012)
Supplement: Supplementary file 1 [file Table_1.DOCX]

**Table S1:** List of viral RNA positive nasopharyngeal swab samples with unique mutations in RBD region, collected from March 2021 to 7^th^ July 2021 and the corresponding accession number in GenBank, NCBI database.

| **Non-Vaccinated** | | | | | | | | |
| --- | --- | --- | --- | --- | --- | --- | --- | --- |
| **Collection Month** | **Sample ID** | **V382L** | **N440K** | **L452R** | **T478K** | **E484Q/K** | **N501Y** | **Accession number** |
| Mar 2021 | CD/21/0746 |  |  | R |  | Q |  | MW969569 |
| Mar 2021 | CD/21/0761 |  |  |  |  | K |  | MW969570 |
| Mar 2021 | CD/21/0772 | L |  | R |  | Q |  | MW969571 |
| Mar 2021 | CD/21/0783 |  |  | R |  | Q |  | MW969752 |
| Mar 2021 | CD/21/0812 |  | K |  |  |  |  | MW969573 |
| Mar 2021 | CD/21/0826 |  |  |  |  |  |  | MW969574 |
| Mar 2021 | CD/21/0871 |  |  | R |  | Q |  | MW969575 |
| Mar 2021 | CD/21/0872 |  |  | R |  | Q |  | MW969576 |
| Mar 2021 | CD/21/0884 |  |  | R |  | K |  | MW969577 |
| Mar 2021 | CD/21/0896 |  | K |  |  |  |  | MW969578 |
| Mar 2021 | CD/21/0899 |  |  | R |  | Q |  | MW969579 |
| Mar 2021 | CD/21/0909 |  |  | R |  | Q |  | MW969580 |
| Mar 2021 | CD/21/0910 |  |  | R |  | Q |  | MW969581 |
| Mar 2021 | CD/21/0912 |  |  | R |  | Q |  | MW969582 |
| Mar 2021 | CD/21/0916 |  |  | R |  | Q |  | MW969583 |
| Mar 2021 | CD/21/0921 |  |  | R |  | Q |  | MW969584 |
| Mar 2021 | CD/21/0922 |  |  |  |  |  |  | MW969585 |
| Mar 2021 | CD/21/0926 |  |  | R |  | Q |  | MW969586 |
| Mar 2021 | CD/21/0927 | L |  | R |  | Q |  | MW969587 |
| Mar 2021 | CD/21/0929 | L |  | R |  | Q |  | MW969588 |
| Mar 2021 | CD/21/1284 |  |  |  |  | Q |  | MW969589 |
| Mar 2021 | CD/21/1286 |  |  | R |  | Q |  | MW969591 |
| Mar 2021 | CD/21/1288 |  |  | R |  | Q |  | MW969593 |
| Mar 2021 | CD/21/1289 |  |  | R | K |  |  | MW969594 |
| Mar 2021 | CD/21/1290 | L |  | R |  | Q |  | MW969595 |
| Mar 2021 | CD/21/1294 |  |  | R |  | Q |  | MW969597 |
| Mar 2021 | CD/21/1295 |  |  |  |  | K |  | MW969603 |
| Mar 2021 | CD/21/1298 |  |  | R |  | Q |  | MW969598 |
| Mar 2021 | CD/21/1299 |  |  |  |  |  |  | MW969599 |
| Mar 2021 | CD/21/1304 |  |  | R |  | Q |  | MW969600 |
| Mar 2021 | CD/21/1305 |  |  | R |  | Q |  | MW969601 |
| Mar 2021 | CD/21/1365 | L |  | R |  | Q |  | MZ577981 |
| Mar 2021 | CD/21/1406 |  |  | R |  | Q |  | MW969604 |
| Apr 2021 | CD/21/1627 |  |  | R | K |  |  | MZ577983 |
| Apr 2021 | CD/21/1628 |  | K |  |  |  |  | MZ577984 |
| Apr 2021 | CD/21/1655 |  |  | R |  | Q |  | MZ577985 |
| Apr 2021 | CD/21/1910 |  |  |  |  |  | Y | MZ577987 |
| Apr 2021 | CD/21/1928 |  |  | R | K |  |  | MZ577988 |
| Apr 2021 | CD/21/2059 |  |  | R | K |  |  | MZ021347 |
| Apr 2021 | CD/21/2062 |  |  | R |  | Q |  | MZ021348 |
| Apr 2021 | CD/21/2063 |  |  | R |  | Q |  | MZ021349 |
| Apr 2021 | CD/21/2064 |  |  | R |  | Q |  | MZ021350 |
| Apr 2021 | CD/21/2066 | L |  | R |  | Q |  | MZ021351 |
| Apr 2021 | CD/21/2073 |  |  | R | K |  |  | MZ021352 |
| Apr 2021 | CD/21/2076 |  |  | R | K |  |  | MZ021353 |
| Apr 2021 | CD/21/2080 |  |  | R | K |  |  | MZ021354 |
| Apr 2021 | CD/21/2081 |  |  | R | K |  |  | MZ021355 |
| Apr 2021 | CD/21/2088 |  |  | R | K |  |  | MZ021356 |
| Apr 2021 | CD/21/2089 |  |  | R |  | Q |  | MZ021357 |
| Apr 2021 | CD/21/2090 | L |  | R |  | Q |  | MZ021358 |
| Apr 2021 | CD/21/2091 |  |  | R |  | Q |  | MZ021359 |
| Apr 2021 | CD/21/2095 |  |  | R | K |  |  | MZ021360 |
| Apr 2021 | CD/21/2096 |  |  | R |  | Q |  | MZ021361 |
| Apr 2021 | CD/21/2098 |  |  | R | K |  |  | MZ021362 |
| Apr 2021 | CD/21/2101 |  |  | R | K |  |  | MZ021363 |
| Apr 2021 | CD/21/2103 |  |  | R | K |  |  | MZ021364 |
| Apr 2021 | CD/21/2104 |  |  | R | K |  |  | MZ021365 |
| Apr 2021 | CD/21/2127 |  |  | R |  | Q |  | MZ674215 |
| Apr 2021 | CD/21/2178 |  |  | R |  | Q |  | MZ577992 |
| Apr 2021 | CD/21/2219 |  |  | R | K |  |  | MZ577993 |
| Apr 2021 | CD/21/2260 | L |  | R |  | Q |  | MZ577990 |
| Apr 2021 | CD/21/2277 |  |  | R |  | Q |  | MZ577991 |
| Apr 2021 | CD/21/2370-2 |  |  |  |  |  |  | MZ577972 |
| Apr 2021 | CD/21/2430 |  |  | R | K |  |  | MZ577994 |
| Apr 2021 | CD/21/2442 |  |  | R | K |  |  | MZ577995 |
| Apr 2021 | CD/21/2552 |  |  | R | K |  |  | MZ577973 |
| Apr 2021 | CD/21/2569 |  |  | R | K |  |  | MZ577829 |
| Apr 2021 | CD/21/2597 |  |  | R | K |  |  | MZ577974 |
| Apr 2021 | CD/21/2605 |  |  | R | K |  |  | MZ577996 |
| Apr 2021 | CD/21/2608 |  |  | R |  | Q |  | MZ676029 |
| Apr 2021 | CD/21/2628-2 |  |  | R | K |  |  | MZ577975 |
| Apr 2021 | CD/21/2685-2 |  |  | R | K |  |  | MZ577976 |
| Apr 2021 | CD/21/2690-2 |  |  | R | K |  |  | MZ674216 |
| Apr 2021 | CD/21/2769 |  |  | R | K |  |  | MZ674217 |
| Apr 2021 | CD/21/2788-2 |  |  | R | K |  |  | MZ577977 |
| Apr 2021 | CD/21/2807 |  |  | R | K |  |  | MZ577978 |
| May 2021 | CD/21/2869-2 |  |  | R | K |  |  | MZ674218 |
| Apr 2021 | CD/21/2957 |  |  | R | K |  |  | MZ674219 |
| Apr 2021 | CD/21/2969 |  |  | R | K |  |  | MZ674220 |
| Apr 2021 | CD/21/2978 |  |  | R | K |  |  | MZ674221 |
| Apr 2021 | CD/21/2999 |  |  | R | K |  |  | MZ674222 |
| Apr 2021 | CD/21/3009 |  |  | R | K |  |  | MZ577979 |
| Apr 2021 | CD/21/3014 |  |  | R | K |  |  | MZ674223 |
| Apr 2021 | CD/21/3028 |  |  | R | K |  |  | MZ577980 |
| Apr 2021 | CD/21/3049 |  |  | R | K |  |  | MZ674224 |
| Apr 2021 | CD/21/3063 |  |  | R | K |  |  | MZ577883 |
| Apr 2021 | CD/21/3064 |  |  | R | K |  |  | MZ674225 |
| Apr 2021 | CD/21/3066 |  |  | R |  | Q |  | MZ577997 |
| May 2021 | CD/21/3136-3 |  |  | R | K |  |  | MZ674226 |
| May 2021 | CD/21/3206 |  |  | R | K |  |  | MZ674228 |
| May 2021 | CD/21/3258 |  |  | R | K |  |  | MZ674229 |
| May 2021 | CD/21/3358 |  |  | R | K |  |  | MZ674230 |
| May 2021 | CD/21/3426 |  |  | R | K |  |  | MZ577958 |
| May 2021 | CD/21/3428 |  |  | R | K |  |  | MZ577951 |
| May 2021 | CD/21/3430 |  |  | R | K |  |  | MZ577952 |
| May 2021 | CD/21/3432 |  |  | R | K |  |  | MZ577953 |
| May 2021 | CD/21/3433 |  |  | R | K |  |  | MZ674231 |
| May 2021 | CD/21/3434 |  |  | R | K |  |  | MZ577954 |
| May 2021 | CD/21/3436 |  |  | R | K |  |  | MZ577955 |
| May 2021 | CD/21/3437 |  |  | R | K |  |  | MZ577956 |
| May 2021 | CD/21/3439 |  |  | R | K |  |  | MZ577957 |
| May 2021 | CD/21/3443 |  |  | R | K |  |  | MZ577959 |
| May 2021 | CD/21/3444 |  |  | R | K |  |  | MZ577960 |
| May 2021 | CD/21/3446 |  |  | R | K |  |  | MZ577961 |
| May 2021 | CD/21/3448 |  |  | R | K |  |  | MZ577962 |
| May 2021 | CD/21/3450 |  |  | R | K |  |  | MZ577963 |
| May 2021 | CD/21/3506 |  |  | R | K |  |  | MZ674232 |
| May 2021 | CD/21/3507 |  |  | R | K |  |  | MZ674233 |
| May 2021 | CD/21/3509 |  |  | R | K |  |  | MZ577964 |
| May 2021 | CD/21/3511 |  |  | R | K |  |  | MZ577965 |
| May 2021 | CD/21/3512 |  |  | R | K |  |  | MZ577966 |
| May 2021 | CD/21/3522 |  |  | R | K |  |  | MZ577934 |
| May 2021 | CD/21/3525 |  |  | R | K |  |  | MZ577936 |
| May 2021 | CD/21/3528 |  |  | R | K |  |  | MZ674331 |
| May 2021 | CD/21/3570 |  |  | R | K |  |  | MZ577937 |
| May 2021 | CD/21/3572 |  |  | R | K |  |  | MZ577938 |
| May 2021 | CD/21/3574 |  |  | R | K |  |  | MZ577939 |
| May 2021 | CD/21/3577 |  |  | R | K |  |  | MZ577940 |
| May 2021 | CD/21/3579 |  |  | R | K |  |  | MZ577941 |
| May 2021 | CD/21/3581 |  |  | R | K |  |  | MZ577942 |
| May 2021 | CD/21/3583 |  |  | R | K |  |  | MZ577943 |
| May 2021 | CD/21/3594 |  |  | R | K |  |  | MZ577944 |
| May 2021 | CD/21/3597 |  |  | R | K |  |  | MZ577945 |
| May 2021 | CD/21/3600 |  |  | R | K |  |  | MZ674332 |
| May 2021 | CD/21/3605 |  |  | R | K |  |  | MZ577926 |
| May 2021 | CD/21/3609 |  |  | R | K |  |  | MZ577946 |
| May 2021 | CD/21/3620 |  |  | R | K |  |  | MZ577949 |
| May 2021 | CD/21/3627 |  |  | R | K |  |  | MZ577927 |
| May 2021 | CD/21/3629 |  |  | R | K |  |  | MZ577928 |
| May 2021 | CD/21/3661 |  |  | R | K |  |  | MZ577929 |
| May 2021 | CD/21/3669 |  |  | R | K |  |  | MZ577930 |
| May 2021 | CD/21/3670 |  |  | R | K |  |  | MZ577931 |
| May 2021 | CD/21/3671 |  |  | R | K |  |  | MZ577932 |
| May 2021 | CD/21/3692 |  |  | R | K |  |  | MZ674333 |
| May 2021 | CD/21/3713 |  |  | R | K |  |  | MZ577831 |
| May 2021 | CD/21/3720 |  |  | R | K |  |  | MZ577832 |
| May 2021 | CD/21/3724 |  |  | R | K |  |  | MZ577833 |
| May 2021 | CD/21/3726 |  |  | R | K |  |  | MZ577834 |
| May 2021 | CD/21/3737 |  |  | R | K |  |  | MZ674334 |
| May 2021 | CD/21/3738 |  |  | R | K |  |  | MZ577835 |
| May 2021 | CD/21/3740 |  |  | R | K |  |  | MZ577836 |
| May 2021 | CD/21/3741 |  |  | R | K |  |  | MZ577837 |
| May 2021 | CD/21/3743 |  |  | R | K |  |  | MZ577838 |
| May 2021 | CD/21/3763-2 |  |  | R | K |  |  | MZ674335 |
| May 2021 | CD/21/3780 |  |  | R | K |  |  | MZ577839 |
| May 2021 | CD/21/3785 |  |  | R | K |  |  | MZ577840 |
| May 2021 | CD/21/3787 |  |  | R | K |  |  | MZ577841 |
| May 2021 | CD/21/3792 |  |  | R | K |  |  | MZ577842 |
| May 2021 | CD/21/3798 |  |  | R | K |  |  | MZ577843 |
| May 2021 | CD/21/3799 |  |  | R | K |  |  | MZ577844 |
| May 2021 | CD/21/3801 |  |  | R | K |  |  | MZ674336 |
| May 2021 | CD/21/3825 |  |  | R | K |  |  | MZ674337 |
| May 2021 | CD/21/3834-2 |  |  | R | K |  |  | MZ674338 |
| May 2021 | CD/21/3866 |  |  | R | K |  |  | MZ674339 |
| May 2021 | CD/21/3890 |  |  | R | K |  |  | MZ674389 |
| May 2021 | CD/21/3892 |  |  | R | K |  |  | MZ674390 |
| May 2021 | CD/21/3909 |  |  | R | K |  |  | MZ674391 |
| May 2021 | CD/21/3967 |  |  | R | K |  |  | MZ674283 |
| May 2021 | CD/21/3968 |  |  | R | K |  |  | MZ674284 |
| May 2021 | CD/21/4061 |  |  | R | K |  |  | MZ674286 |
| Jun 2021 | CD/21/4053-2 |  |  | R | K |  |  | MZ674285 |
| Jun 2021 | CD/21/4127 |  |  | R | K |  |  | MZ674288 |
| Jun 2021 | CD/21/4198-2 |  |  | R | K |  |  | MZ674289 |
| Jun 2021 | CD/21/4267 |  |  | R | K |  |  | MZ674291 |
| Jun 2021 | CD/21/4296 |  |  |  |  |  |  | MZ577858 |
| Jun 2021 | CD/21/4317 |  |  | R | K |  |  | MZ674292 |
| Jun 2021 | CD/21/4331 |  |  | R | K |  |  | MZ674293 |
| Jun 2021 | CD/21/4345-2 |  |  | R | K |  |  | MZ674294 |
| **Vaccinated- 1 dose** | | | | | | | | |
| Mar 2021 | CD/21/1285 | L |  | R |  | Q |  | MW969590 |
| Mar 2021 | CD/21/1287 |  |  | R |  | Q |  | MW969592 |
| Mar 2021 | CD/21/1291 | L |  | R |  | Q |  | MW969596 |
| Mar 2021 | CD/21/1292 |  |  | R |  | Q |  | MZ676028 |
| Mar 2021 | CD/21/1306 |  |  | R |  | Q |  | MW969602 |
| Mar 2021 | CD/21/1326 |  |  |  |  |  |  | MZ577813 |
| Mar 2021 | CD/21/1340 |  |  | R |  | Q |  | MZ577814 |
| Mar 2021 | CD/21/1431 |  |  |  |  |  |  | OM135253 |
| Mar 2021 | CD/21/1432 |  |  | R |  | Q |  | MZ577982 |
| Mar 2021 | CD/21/1488 |  |  | R |  | Q |  | MZ577815 |
| Apr 2021 | CD/19/1059-4 |  |  | R | K |  |  | MZ577845 |
| Apr 2021 | CD/21/1605-2 |  |  | R | K |  |  | MZ577846 |
| Apr 2021 | CD/21/1643 |  |  | R | K |  |  | MZ577818 |
| Apr 2021 | CD/21/1656 |  |  | R | K |  |  | MZ577971 |
| Apr 2021 | CD/21/1703 |  |  | R |  | Q |  | MZ577879 |
| Apr 2021 | CD/21/1721 |  |  | R |  | Q |  | MZ577986 |
| Apr 2021 | CD/21/1726 |  |  | R |  | Q |  | MZ577878 |
| Apr 2021 | CD/21/1789 |  |  | R | K |  |  | MZ577847 |
| Apr 2021 | CD/21/1791 |  |  | R |  | Q |  | MZ577848 |
| Apr 2021 | CD/21/1793 |  |  | R |  | Q |  | MZ577849 |
| Apr 2021 | CD/21/1811 |  |  | R |  | Q |  | MZ577850 |
| Apr 2021 | CD/21/1812 |  |  | R |  | Q |  | MZ577851 |
| Apr 2021 | CD/21/1813 |  |  | R |  | Q |  | MZ577852 |
| Apr 2021 | CD/21/1816 |  |  | R |  | Q |  | MZ577854 |
| Apr 2021 | CD/21/1820 |  |  | R |  | Q |  | MZ577820 |
| Apr 2021 | CD/21/1856 |  |  | R |  | Q |  | MZ577821 |
| Apr 2021 | CD/21/1894 |  |  | R |  | Q |  | MZ577822 |
| Apr 2021 | CD/21/1901 |  |  | R |  | Q |  | MZ577824 |
| Apr 2021 | CD/21/1905 |  |  | R |  | Q |  | MZ577825 |
| Apr 2021 | CD/21/1907 |  |  | R |  | Q |  | MZ577827 |
| Apr 2021 | CD/21/1932 | L |  | R | K |  |  | MZ577895 |
| Apr 2021 | CD/21/1933 |  |  | R | K |  |  | MZ577828 |
| Apr 2021 | CD/21/1941 |  |  | R | K |  |  | MZ577856 |
| Apr 2021 | CD/21/2130 |  |  | R | K |  |  | MZ577857 |
| Apr 2021 | CD/21/2175 |  |  | R | K |  |  | MZ577989 |
| Apr 2021 | CD/21/3057 |  |  | R | K |  |  | MZ577896 |
| Apr 2021 | CD/21/3086 |  |  | R | K |  |  | MZ577897 |
| Apr 2021 | CD/21/3089 |  |  | R | K |  |  | MZ577898 |
| Apr 2021 | CD/21/3124 |  |  | R | K |  |  | MZ577875 |
| May 2021 | CD/21/3162 |  |  | R | K |  |  | MZ577914 |
| May 2021 | CD/21/3169 |  |  | R | K |  |  | MZ577915 |
| May 2021 | CD/21/3174 |  |  | R | K |  |  | MZ577916 |
| May 2021 | CD/21/3179 |  |  | R | K |  |  | MZ577917 |
| May 2021 | CD/21/3261 |  |  | R | K |  |  | OM135254 |
| May 2021 | CD/21/3362 |  |  | R | K |  |  | MZ577899 |
| May 2021 | CD/21/3366 |  |  | R | K |  |  | MZ577900 |
| May 2021 | CD/21/3378 |  |  | R |  | Q |  | MZ577901 |
| May 2021 | CD/21/3427 |  |  | R | K |  |  | MZ577950 |
| May 2021 | CD/21/3518 |  |  | R | K |  |  | MZ577967 |
| May 2021 | CD/21/3523 |  |  | R | K |  |  | MZ577935 |
| May 2021 | CD/21/3561-2 |  |  | R | K |  |  | MZ577877 |
| May 2021 | CD/21/3562-2 |  |  | R | K |  |  | MZ676027 |
| May 2021 | CD/21/3602 |  |  | R | K |  |  | MZ577921 |
| May 2021 | CD/21/3613 |  |  | R | K |  |  | MZ577948 |
| May 2021 | CD/21/3626 |  |  | R | K |  |  | MZ577902 |
| May 2021 | CD/21/3683 |  |  | R | K |  |  | MZ577830 |
| May 2021 | CD/21/3704 |  |  | R |  | Q |  | MZ676030 |
| May 2021 | CD/21/3706 |  |  | R | K |  |  | MZ577906 |
| May 2021 | CD/21/3707 |  |  | R |  | Q |  | MZ577907 |
| May 2021 | CD/21/3715 |  |  | R | K |  |  | MZ577923 |
| May 2021 | CD/21/3721 |  |  | R | K |  |  | MZ577924 |
| May 2021 | CD/21/3769 |  |  | R | K |  |  | MZ577870 |
| May 2021 | CD/21/3776 |  |  | R | K |  |  | MZ577872 |
| May 2021 | CD/21/3796 |  |  | R | K |  |  | MZ577909 |
| May 2021 | CD/21/3865 |  |  | R | K |  |  | MZ676031 |
| May 2021 | CD/21/3874 |  |  | R | K |  |  | MZ676032 |
| May 2021 | CD/21/4067 |  |  | R | K |  |  | MZ577888 |
| May 2021 | CD/21/3893 |  |  | R | K |  |  | MZ577910 |
| May 2021 | CD/21/3941 |  |  | R | K |  |  | MZ577913 |
| May 2021 | CD/21/4044 |  |  | R | K |  |  | MZ577882 |
| May 2021 | CD/21/4065 |  |  | R | K |  |  | MZ577884 |
| May 2021 | CD/21/4068 |  |  | R | K |  |  | MZ577891 |
| May 2021 | CD/21/4069 |  |  | R | K |  |  | MZ577885 |
| May 2021 | CD/21/4074 |  |  | R | K |  |  | MZ577886 |
| May 2021 | CD/21/4075 |  |  | R | K |  |  | MZ577893 |
| May 2021 | CD/21/4078 |  |  | R | K |  |  | MZ577889 |
| May 2021 | CD/21/4088 |  |  | R | K |  |  | MZ577894 |
| May 2021 | CD/21/4096 |  |  | R | K |  |  | OM135258 |
| May 2021 | CD/21/4117 |  |  | R | K |  |  | MZ674287 |
| Jun 2021 | CD/21/4136 |  |  | R | K |  |  | OM135260 |
| Jun 2021 | CD/21/4173 |  |  | R | K |  |  | OM135263 |
| Jun 2021 | CD/21/4179-2 |  |  | R | K |  |  | OM135265 |
| Jun 2021 | CD/21/4185-2 |  |  | R | K |  |  | OM135267 |
| Jun 2021 | CD/21/4276-2 |  |  | R | K |  |  | OM135268 |
| **Vaccinated- 2 doses** | | | | | | | | |
| Mar 2021 | CD/21/1373 |  |  | R |  | Q |  | MZ577968 |
| Mar 2021 | CD/21/1374 |  |  | R |  | Q |  | MZ577969 |
| Mar 2021 | CD/21/1494 |  |  | R | K |  |  | MZ577816 |
| Apr 2021 | CD/21/1623 |  |  | R |  | Q |  | MZ577970 |
| Apr 2021 | CD/21/1629 | L |  | R |  | Q |  | MZ577817 |
| Apr 2021 | CD/21/1815 |  |  | R | K |  |  | MZ577853 |
| Apr 2021 | CD/21/1817 |  |  | R |  | Q |  | MZ577855 |
| Apr 2021 | CD/21/1844 |  |  | R | K |  |  | MZ577819 |
| Apr 2021 | CD/21/1898 |  |  | R |  | Q |  | MZ577823 |
| Apr 2021 | CD/21/1906 |  |  | R | K |  |  | MZ577826 |
| Apr 2021 | CD/21/3122 |  |  | R | K |  |  | MZ577876 |
| May 2021 | CD/21/3210 |  |  | R | K |  |  | MZ577918 |
| May 2021 | CD/21/3588 |  |  | R | K |  |  | MZ577919 |
| May 2021 | CD/21/3595 |  |  | R | K |  |  | MZ577920 |
| May 2021 | CD/21/3611 |  |  | R | K |  |  | MZ577947 |
| May 2021 | CD/21/3679 |  |  | R |  | Q |  | MZ577933 |
| May 2021 | CD/21/3702 |  |  | R | K |  |  | MZ577903 |
| May 2021 | CD/21/3703 |  |  | R | K |  |  | MZ577904 |
| May 2021 | CD/21/3705 |  |  | R | K |  |  | MZ577905 |
| May 2021 | CD/21/3708 |  |  | R | K |  |  | MZ597836 |
| May 2021 | CD/21/3710 |  |  | R |  | Q |  | MZ577908 |
| May 2021 | CD/21/3711 |  |  | R |  | Q |  | MZ577922 |
| May 2021 | CD/21/3770 |  |  | R | K |  |  | MZ577873 |
| May 2021 | CD/21/3771 | L |  | R |  | Q |  | MZ577871 |
| May 2021 | CD/21/3777 | L |  | R |  | Q |  | MZ577874 |
| May 2021 | CD/21/3861 |  |  | R |  | Q |  | MZ577925 |
| May 2021 | CD/21/3918 |  |  | R | K |  |  | MZ577911 |
| May 2021 | CD/21/3923 |  |  | R |  | Q |  | MZ577912 |
| May 2021 | CD/21/4026 |  |  | R | K |  |  | MZ577881 |
| May 2021 | CD/21/4070 |  |  | R | K |  |  | MZ577890 |
| May 2021 | CD/21/4073 |  |  | R | K |  |  | MZ577892 |
| May 2021 | CD/21/4077 |  |  | R | K |  |  | MZ577887 |
| May 2021 | CD/21/4090 |  |  | R | K |  |  | OM135262 |
| Jun 2021 | CD/21/4101 |  |  | R | K |  |  | OM135261 |
| Jun 2021 | CD/21/4212-2 |  |  | R | K |  |  | MZ674290 |
| Jun 2021 | CD/21/4238-2 |  |  | R | K |  |  | OM135264 |
| Jun 2021 | CD/21/4287-2 |  |  | R | K |  |  | OM135257 |
| Jun 2021 | CD/21/4323-2 |  |  | R | K |  |  | OM135255 |
| Jun 2021 | CD/21/4357-3 |  |  | R | K |  |  | OM135269 |
| Jul 2021 | CD/21/4457 |  |  | R | K |  |  | OM135256 |
| Jul 2021 | CD/21/4470 |  |  | R | K |  |  | OM135266 |
| Jul 2021 | CD/21/4477 |  |  | R | K |  |  | OM135259 |
